# Three-Dimensional Heterostructured Reduced Graphene Oxide-Hexagonal Boron Nitride-Stacking Material for Silicone Thermal Grease with Enhanced Thermally Conductive Properties

**DOI:** 10.3390/nano9070938

**Published:** 2019-06-28

**Authors:** Weijie Liang, Xin Ge, Jianfang Ge, Tiehu Li, Tingkai Zhao, Xunjun Chen, Mingchang Zhang, Jianye Ji, Xiaoyan Pang, Ruoling Liu

**Affiliations:** 1 Shaanxi Engineering Laboratory of Graphene New Carbon Materials and Applications, School of Materials Science and Engineering, Northwestern Polytechnical University, Xi’an 710072, China; 2 Guangdong Engineering Research Center of Silicone Electronic Fine Chemicals, College of Chemistry and Chemical Engineering, Zhongkai University of Agriculture and Engineering, Guangzhou 510225, China

**Keywords:** reduced graphene oxide, hexagonal boron nitride, silicone thermal grease, viscosity, thermally conductive properties

## Abstract

The thermally conductive properties of silicone thermal grease enhanced by hexagonal boron nitride (hBN) nanosheets as a filler are relevant to the field of lightweight polymer-based thermal interface materials. However, the enhancements are restricted by the amount of hBN nanosheets added, owing to a dramatic increase in the viscosity of silicone thermal grease. To this end, a rational structural design of the filler is needed to ensure the viable development of the composite material. Using reduced graphene oxide (RGO) as substrate, three-dimensional (3D) heterostructured reduced graphene oxide-hexagonal boron nitride (RGO-hBN)-stacking material was constructed by self-assembly of hBN nanosheets on the surface of RGO with the assistance of binder for silicone thermal grease. Compared with hBN nanosheets, 3D RGO-hBN more effectively improves the thermally conductive properties of silicone thermal grease, which is attributed to the introduction of graphene and its phonon-matching structural characteristics. RGO-hBN/silicone thermal grease with lower viscosity exhibits higher thermal conductivity, lower thermal resistance and better thermal management capability than those of hBN/silicone thermal grease at the same filler content. It is feasible to develop polymer-based thermal interface materials with good thermal transport performance for heat removal of modern electronics utilising graphene-supported hBN as the filler at low loading levels.

## 1. Introduction

Facing the trend of continuing miniaturisation and high-power densification of microelectronics, strategies and techniques for more efficient heat removal are becoming increasingly desirable and necessary in order to help develop new innovative techniques and technologically advance in the modern electronics industry. Thermal interface materials have played an important role in terms of thermal management due to their heat conducting capabilities. These materials can be categorised into several types according to different properties and applications [1]. In view of their easy processing, light weight, inexpensiveness and excellent electrical insulation, considerable interest has been focused on polymer-based thermal interface materials [2,3,4]. Silicone thermal grease is a typical polymer-based thermal interface material containing silicone and thermal conductive fillers, usually used to take the place of air and bond the jointed solid contact surfaces of heat sink and devices so as to dissipate heat. Significant improvement of thermally conductive properties mainly relies on the loading of fillers, such as ceramics [5], metals [6], carbon materials [7] and their hybrid particles [8].

Hexagonal boron nitride (hBN), a honeycomb configuration of sp^2^-bonded boron and nitrogen, has exhibited various advantages owing to its distinct structural properties, such as lightweight, anisotropic thermal conductivity and electric insulation. The in-plane and out-of-plane thermal conductivity of hBN is 600 and 30 W·m^−1^·K^−1^, respectively [9]. These prominent properties made it suitable for preparing polymer-based thermal interface material as thermally conductive filler. The reported micro-sized-hBN-filled polymer-based thermal interface materials show high thermal conductivity with a high loading of fillers [10,11,12]. However, unlike micro-sized-hBN, it is difficult to fill amounts of nano-sized hBN sheets into silicone thermal grease because of a sharp increase in viscosity. The hyperviscosity of silicone thermal grease goes against not only processing during the course of fabrication, but also installing in the device for application. As reported previously [13], the differences in the shape and size of fillers have different effects in the viscosity of polymer composites. For instance, Ren et al. [14] found that the large-size spherical hBN did not easily increase the viscosity of pre-cured polymer matrix compared with the small-size platelet-like hBN in the high filler content region. Therefore, hBN nanosheets can be considered to assemble themselves into a larger and unique shape filler in order to overcome the restriction from viscosity.

Graphene, a two-dimensional (2D) carbon hexagonal lattice, has been regarded as an excellent thermal conductor. The covalent sp^2^ conjugated bonding between carbon atoms of graphene brings about an extraordinarily high thermal conductivity (~5300 W·m^−1^·K^−1^). Graphene has been widely studied as an additive for polymer-based thermal interface material. Great enhancements have been observed in the thermally conductive properties of different polymer matrices with a low loading of graphene [15,16]. To make use of synergistic effect to increase thermal conductivity and improve other performances of a polymer-based thermal interface, much research has been done to hybridise graphene with other thermal conductive fillers including hBN [17], SiC [18], Al_2_O_3_ [19], Ag [20], carbon nanotubes [21] and so on. For example, Yao et al. [17] fabricated a 3D skeleton by assembling micrometre-sized hBN and reduced graphene oxide (RGO) in epoxy resin using ice-templated assembly technology and a vacuum-assisted infiltration method. This presented excellent thermal management capacity and electrical insulation. In addition, some studies have suggested that graphene is a potential phonon-transferring substrate for hBN due to the small lattice mismatch, which is good for high heat dissipation [22,23,24].

Combining the advantages of both graphene and hBN may be an appealing and promising alternative for silicone thermal grease with enhanced thermally conductive properties. Here, a simple new method is developed to obtain hybrid material composed of RGO and hBN nanosheets. Three-dimensional heterostructured reduced graphene oxide–hexagonal boron nitride-stacking material labelled as RGO-hBN was fabricated by self-assembly of hBN nanosheets on the surface of RGO with the assistance of polyvinyl alcohol (PVA) as a binder. The detailed synthesis steps, as well as both the morphology and structure of the as-prepared RGO-hBN, are presented. The RGO-hBN was introduced into a market-available silicone thermal grease (STG) to form RGO-hBN/STG composites. The viscosity and thermally conductive properties of the composites were investigated.

## 2. Materials and Methods 

### 2.1. Materials

Hexagonal boron nitride (hBN, 500 nm) was made in HAOXI Research Nanomaterials, Inc. (Shanghai, China). The reduced graphene oxide (RGO) was fabricated according to our previous work [25]. The binder polyvinyl alcohol (PVA, BP-17) was produced by Chang Chun Chemical (Jiangsu) Co., Ltd. (Changshu, China). The dispersant sodium dodecylbenzenesulfonate (SDBS, AR) was purchased from Shanghai Aladdin Biochemical Technology Co., Ltd. (Shanghai, China). Silicone thermal grease (STG, HN-120) mainly containing dimethicone (500 mPa·s) and thermal conductive fillers (ZnO and Al_2_O_3_) was provided by Zhongshan Huineng Silicone Co., Ltd. (Zhongshan, China).

### 2.2. Synthesis of RGO-hBN

The RGO-hBN was synthesised by a facile method as follows. Firstly, 2 g hBN and 100 mg RGO were added into 500 mL deionised water containing a small amount of dispersant SDBS by ultrasonication for 5 h. Then 210 mg 10 wt.% PVA solution was dripped into above dispersion under stirring for 1 h at 60 °C. The mixture was filtered over microfiltration membrane (1.2 μm) and washed with deionised water. It was dried at 85 °C in a vacuum oven for 24 h, and then ground by ball grinding technology in a omni-directional planetary ball mill at 500 rpm for 8 h. Afterwards, the powders was transferred into a quartz tube reactor in argon at 1200 °C for 2 h to remove organic additives and obtain sintered 3D heterostructured RGO-hBN. Finally, the RGO-hBN was collected for further application.

### 2.3. Preparation of RGO-hBN/STG

**Figure 1:**
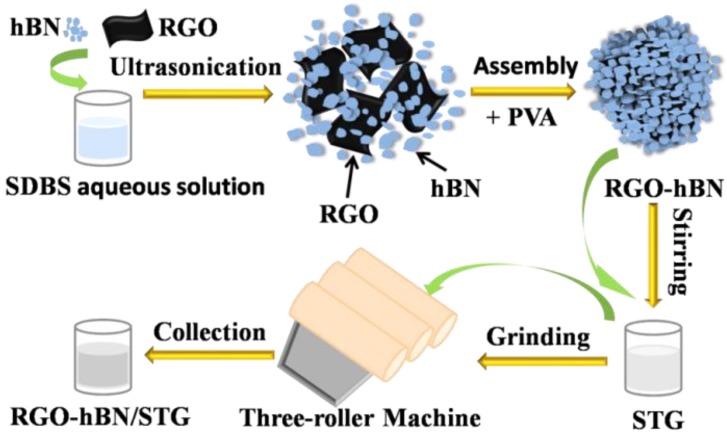
Scheme for the preparation of RGO-hBN/STG.

A STG available on the market was used as the mother material. Using an in situ blending method, RGO-hBN with a weight fraction ranging between 3 and 12 wt.% was compounded with a measured quantity (50 g) of STG controlled with a three-roller machine at room temperature. The milling process was repeated 6–9 times until RGO-hBN was well dispersed in the STG and the as-prepared RGO-hBN/STG became homogeneous. Figure 1 provides the main preparation process for RGO-hBN/STG. STGs with different loadings of hBN nanosheets were prepared by the same procedure for comparison.

### 2.4. Characterisation

A field emission scanning electron microscope (SEM, S4800 and SU8220, Hitachi, Tokyo, Japan), X-ray diffraction patterns (XRD, D8 ADVANCE, Bruker AXS, Karlsruhe, Germany) and Raman scattering spectrum (λ = 532 nm Ar Laser, LabRAM HR800, HORIBA Scientific, Lat Krabang, Thailand) were employed to qualitatively analyse the surface morphology, structural characteristics and components composition of RGO, hBN and RGO-hBN. The dynamic viscosity of RGO-hBN/STG and hBN/STG were measured by a rotational rheometer (AR-1500ex, TA Instruments, New Castle, DE, USA) with the shear rate of 5 1/s at 25 °C. Thermal conductivity of RGO-hBN/STG and hBN/STG was examined by a universal thermal conductivity meter (TC3000, Xiatech, Xi’an, China) using the transient hot-wire method at room temperature. Thermal resistance of RGO-hBN/STG and hBN/STG was tested by a thermal resistance and conductivity measurement apparatus (LW-9389, Longwin, Taoyuan, Taiwan) based on ASTM D 5470-06 Standard with heating temperature of 80 °C, pressure of 50 Psi, and the dimension of specimens was 25.4 × 25.4 × 0.1 mm^3^. Thermal resistance R (°C/W) was obtained by the following equation: R = (T_h_ − T_c_)/Q_ave_, where T_h_ is hot surface temperature (°C), T_c_ is cold surface temperature (°C), and Q_ave_ is average heat flux (W). The thermal management capability of RGO-hBN/STG, hBN/STG and STG were performed by thermal infrared camera (TiS10, Fluke, Madison, WI, USA). Firstly, the hot-stage (K3000-B, Mshot, Guangzhou, China) was heated to 90 °C and maintained at this temperature for testing. Then, a glass uniformly coated with the sample was put on the hot-stage for 5 min. After that, it was removed from the hot-stage and put on the round plate to cool down at ambient temperature. Finally, images were captured every five seconds for recording the change of temperature of the samples in five minutes during the heating and cooling process by thermal infrared camera.

## 3. Results and Discussion

### 3.1. Morphology and Structure of RGO-hBN

**Figure 2:**
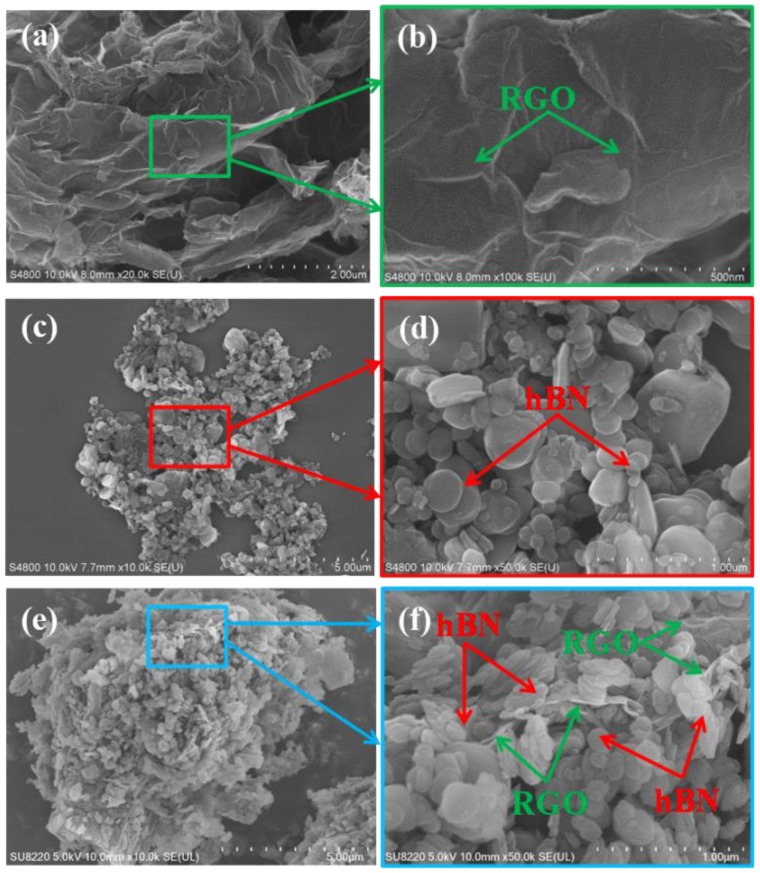
SEM images of (**a**,**b**) RGO, (**c**,**d**) hBN and (**e**,**f**) RGO-hBN at different magnifications.

The morphologies of RGO, hBN and RGO-hBN at different magnifications are shown in Figure 2. As shown in Figure 2a, the bare RGO exhibits a fluffy and multilayer structure after the thermal reduction process which means that graphene can be easily exfoliated via the next step of ultrasonic dispersion. Figure 2b is the high-magnification SEM picture of the marked rectangular region of RGO. It shows the folds on the surface of RGO. The graphene folds are beneficial for the agglomeration of small particles. Figure 2c is the SEM image of pure hBN, displaying the aggregation among nanoparticles. It can be seen that hBN has a smooth surface and perfect sheet nanostructure as shown in Figure 2d. The lateral sizes of the majority of hBN nanosheets are around 500 nm. These hybrid sizes in hBN nanosheets can contribute to preparation of 3D RGO-hBN stacking material for the thermal management application. Figure 2e shows the 3D stack structure of RGO-hBN nanocomposite. The lateral size of RGO-hBN is about 11 μm. This shape and size of particle can be a fine choice for improving thermal conductivity of polymer-based thermal interface materials. Figure 2f is an enlarged figure of the rectangular zone in Figure 2e. Clearly, the RGO-hBN hybrid consists of wrinkled RGO, hBN nanosheets (marked by arrows). The hBN nanosheets bond together to form a cluster configuration assisted by PVA as a binder. The hBN clusters adhere to the surface of RGO densely and directly because of the binder, as well. The adhesion orientation is anisotropic. Graphene, as the internal skeleton, is wrapped in hBN clusters to form a relatively stable 3D RGO-hBN stack architecture. This demonstrates that the 3D RGO-hBN stack structure with RGO between hBN sheets has been fabricated successfully.

**Figure 3:**
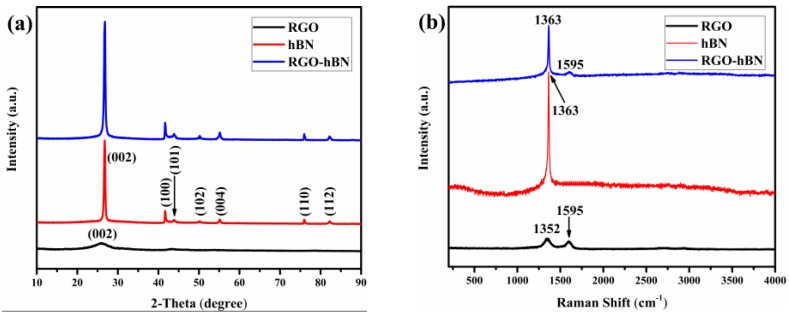
(**a**) XRD patterns and (**b**) Raman spectra of RGO, hBN and RGO-hBN.

The XRD patterns of the hBN nanosheets adhered on the surface of the RGO substrate are displayed in Figure 3a. The main broad diffraction peak of RGO appears at 25.9°, which corresponds to the (002) plane of carbon (C). This is typical of multilayered graphene after high temperature deoxidisation. As for the hBN, its characteristic diffraction peaks at 2θ = 26.8°, 41.6°, 43.9°, 50.1°, 55.1°, 76.0° and 82.2° can be indexed to (002), (100), (101), (102), (004), (110) and (112) of hBN (JCPDS No.34-0421), respectively [26,27]. In comparison with hBN, the XRD pattern of RGO-hBN is similar to that of hBN, without the carbon peak of graphene, which might be due to the fact that the quantity of RGO is far less than that of hBN, so that the characteristic diffraction peak of (002) in hBN overlaps with that which originated from RGO. However, on the one hand, the intensity of the (002) peak of the spectrum of RGO-hBN increases markedly, and its position shifted slightly from 26.8° to 27.0°. On the other hand, the intensity of the other peaks of RGO-hBN are a little higher than that of hBN. The changes can be explained by the size of particles [28]. These results give evidence of the formation of large particles with a stack structure of hBN nanosheets coating the surface of RGO thanks to PVA.

The structure of RGO-hBN is further confirmed by the Raman spectra (Figure 3b). Two characteristic peaks at 1352 cm^−1^ (D band) and 1595 cm^−1^ (G band) can be seen from the curve of RGO, respectively [25]. The peaks seem weak in the figure, because their intensity is much lower than that of hBN. For pure hBN, a sharp and strong peak is observed at 1363 cm^−1^, signifying the high quality of as-used hBN, which involves the intra layer E_2g_ vibration mode of hBN [10,29]. The Raman spectrum of the RGO-hBN shows peaks derived from the vibrational features of hBN and graphene. The bands located at 1363 cm^−1^ and 1595 cm^−1^ match well with the vibration of B-N and sp^2^-hybridised carbon, respectively. The intensity has declined at the same time compared with that of hBN and RGO, manifesting that the components are combined well. However, it is difficult to identify the D band of graphene from the line of RGO-hBN. The reason for this is that the proportion of RGO is small and the position of D band is close to that of the typical peaks of hBN. The Raman results further prove that the 3D RGO-hBN hybrid has been successfully synthesised due to the bonding effect by PVA.

### 3.2. Rheological Behaviour of RGO-hBN/STG

**Figure 4:**
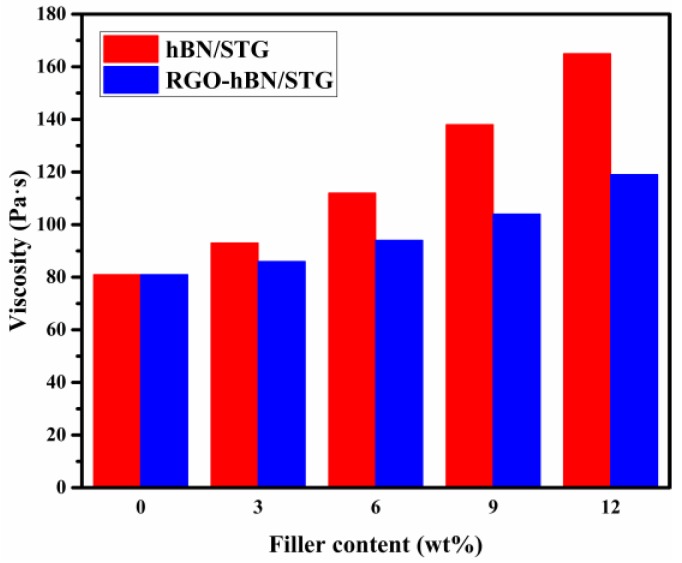
Viscosity of hBN/STG and RGO-hBN/STG with different filler contents.

Rheological behaviour is an issue of common concern for silicone thermal grease, both in processing and application. Low viscosity ensures the feasibility of the processability and constructability. To compare the effect of RGO-hBN and hBN, investigations of viscosity of RGO-hBN/STG and hBN/STG were conducted at a shear rate of 5 1/s and 25 °C, as shown in Figure 4. Without RGO-hBN or hBN, the as-used STG is a composite containing dimethicone, ZnO and Al_2_O_3_, and its viscosity is about 81 Pa·s, showing the typical characteristic of slurry. The viscosity value of STG warrants the loading of fillers for enhancing thermally conductive properties further. The RGO-hBN/STG and hBN/STG showed a continuous increase in viscosity with the increasing of filler content. The RGO-hBN/STG of growth in the scope of viscosity is less than that of hBN/STG in the experimental range. At the filler content of 12 wt.%, the viscosity of the micron-sized 3D heterostructured and stacked RGO-hBN filled mixture was 119 Pa·s, but that of mixture filled with nano-sized platelet-like BN reached as high as 165 Pa·s. Although the viscosity of the former increased compared to STG, the constructability is acceptable in application. However, the viscosity of the latter became too high, and thus the latter deformed, making it difficult to conform the topographies of the mating surfaces. The viscosity in the slurry system is susceptible to changes in the shape and size of particles due to friction resulting from particle–particle interactions [13,14]. Much of the growth in viscosity of hBN/STG can be put down to the high contact area and scattering of hBN nanosheets, which increase the interior friction with ZnO and Al_2_O_3_ of the slurry system. In contrast to hBN nanosheets, the micron-sized RGO-hBN with a 3D stacked structure fares well in reducing the interior friction with other fillers in silicone thermal grease due to its low contact area. As for viscosity, it is proved that RGO-hBN is suitable for silicone thermal grease at a given filler content instead of hBN.

### 3.3. Thermally Conductive Properties of RGO-hBN/STG

**Figure 5:**
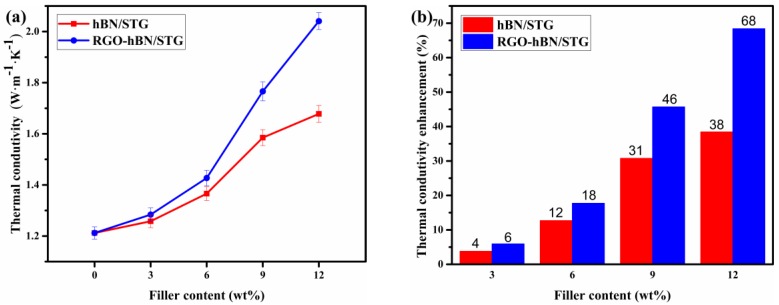
(**a**) Thermal conductivity of hBN/STG and RGO-hBN/STG with different filler contents, and (**b**) the corresponding thermal conductivity enhancement.

Thermal conductivity is one of the general thermally conductive properties of silicone thermal grease. Figure 5a,b shows the variations of the thermal conductivity and their corresponding enhancement with weight contents of RGO-hBN and hBN. The efficiency of the fillers in STG was calculated by the thermal conductivity enhancement according to the following equation [30,31]: η (%) = (K − K_m_)/K_m_ × 100, where K and K_m_ are the thermal conductivity of the corresponding composites (RGO-hBN/STG and hBN/STG) and STG, respectively. Before adding RGO-hBN or hBN, the thermal conductivity of STG with dimethicone, ZnO and Al_2_O_3_ is about 1.21 W·m^−1^·K^−1^ at room temperature. It can be seen that the thermal conductivity of RGO-hBN/STG and hBN/STG increased with the loading of the fillers. Moreover, for the same filler fraction, it can be seen that the thermal conductivity of RGO-hBN/STG is higher than that of hBN/STG. There is a slight increase in the thermal conductivity of RGO-hBN/STG and hBN/STG when the contents are increased from 0 to 3 wt.% (around 5% enhancement), which may be due to the fact that the quality of thermally conductive pathways has not changed much in the system. The thermal conductivity obviously increased from 1.43 to 2.04 W·m^−1^·K^−1^ (about a 18–68% enhancement) when the RGO-hBN content was increased from 6 to 12 wt.%. Thereby, the gap of the thermal conductivity enhancement between RGO-hBN/STG and hBN/STG widened in the experimental range. The enhanced ability of RGO-hBN reached 68%, about 1.8 times that of the hBN in filled STG (38%) at a filling ratio of 12 wt.%. The RGO-hBN is more effective filler than pure h-BN for the enhancement of thermal conductivity, which can be ascribed to the incorporation of RGO. The 3D RGO-hBN stack structure makes the interaction between RGO and hBN stronger, as well as increasing the synergising effect of the components for heat conduction based on the clustering mechanism [32,33]. Importantly, phonon spectra matching of the assembly RGO and hBN is beneficial to promote phonon conduction, which can enhance the thermal conduction performance of the filler [17,23,24]. According to Zhou’s research, the enhancement in the thermal conductivity of the polymer-based thermal interface material with increasing particles size can be due to the formation of effective heat-conductive pathways for the larger particles [34]. Thus, the size of RGO-hBN could be advantageous for forming continuous and stable thermal conductance paths with ZnO and Al_2_O_3_ in the system than raw hBN. In other words, the stack structure and dimensions of RGO-hBN contribute to the enhancement of thermal conductivity.

**Figure 6:**
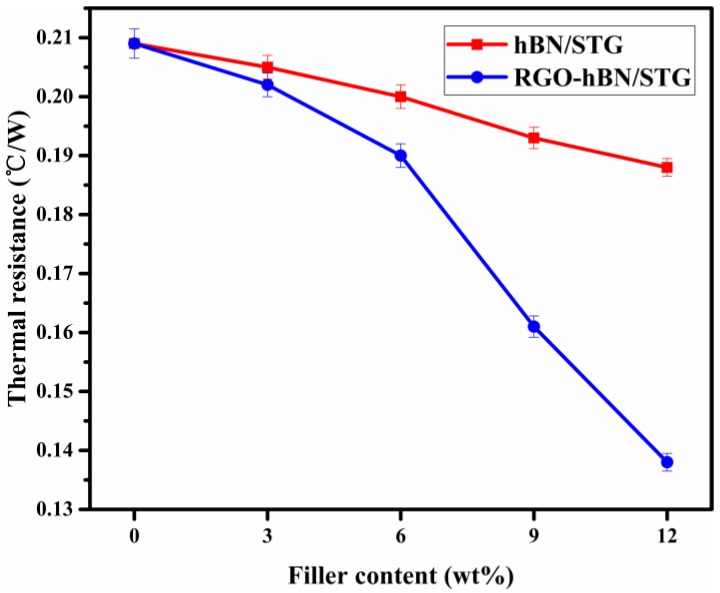
Thermal resistance of hBN/STG and RGO-hBN/STG with different filler contents.

The actual thermal resistance is also an important thermally conductive performance index of silicone thermal grease. Figure 6 depicts the variations of the thermal resistance for RGO-hBN/STG and hBN/STG as a function of filler content. The thermal resistance of the original STG was 0.209 °C/W. If thermal resistance decreases, heat conduction is more effective. The loading of thermal conductive fillers, such as RGO-hBN and h-BN in STG is expected to induce a debasement in its thermal resistance. Indeed, the thermal resistance of RGO-hBN/STG and hBN/STG have the same trend and decrease with the addition of both fillers in different weight content. This highlights the role of RGO-hBN and hBN, which can serve as thermally conductive “bridges” or heat inter-connectors with ZnO and Al_2_O_3_ in the polymer chain [35]. It is proposed here that new and effective thermal conductivity paths were built up to reduce interfacial thermal resistance [36]. The RGO-hBN/STG with RGO-hBN of 12 wt.% showed lower values of thermal resistance, with reduction to as little as 34% when compared with pristine STG. Meanwhile, it is also observed that RGO-hBN significantly outperforms its h-BN counterparts. The drop in thermal resistance of the RGO-hBN/STG is greater than that observed for hBN/STG with the same percentage content of fillers. Specifically, the thermal resistance value of the RGO-hBN/STG decreases to a value of 0.138 °C/W (12 wt.%), lower than 0.188 °C/W of the hBN/STG. These results are attributed mainly to the combination of RGO and hBN nanosheets. The stable 3D stack architecture that they form significantly impacts thermal transport in RGO-supported hBN arising from strong interaction and good phonon spectral matching [17]. Compared to hBN, RGO-hBN and the host fillers of STG can form more favourable thermal conductivity paths and better interface interaction with polymer matrix. The size of RGO-hBN is much bigger than hBN, which has positive effects on the STG, because the thermal resistance of the polymer-based thermal interface material is inversely proportional to filler size [7]. Therefore, the RGO-hBN is more suitable for the reduction in thermal resistance of STG, benefitting from the architecture and size of particle compared with single hBN.

**Figure 7:**
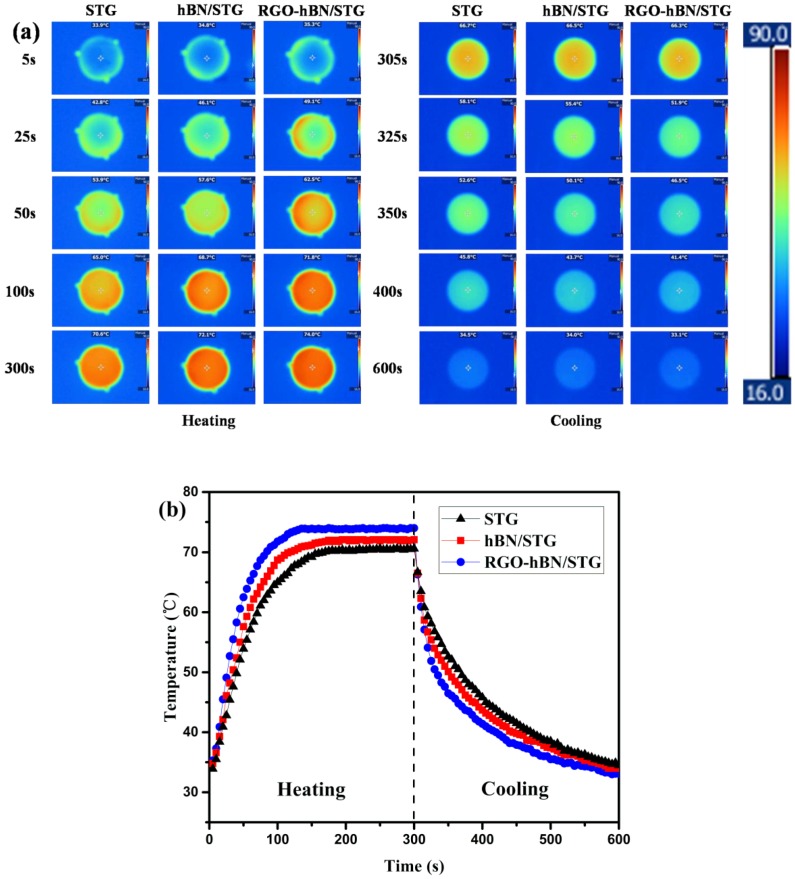
(**a**) Images recorded by a thermal imager with specimens heated on a homothermal platform and (**b**) the surface centre temperature profiles of the samples as function of heating time.

Infrared thermal imaging technique can be employed to evaluate the thermal management capability of silicone thermal grease directly. RGO-hBN/STG with RGO-hBN of 12 wt.%, hBN/STG with hBN of 12 wt.% and STG were subjected to courses of heating and cooling. The surface temperature variations of them with time were monitored by an infrared thermal imager, as shown in Figure 7. To investigate the heat absorption performance, each sample was placed on the hot-stage (90 °C) for 5 min. Detailed temperature distribution images can be observed during heating process from Figure 7a. It is obvious that RGO-hBN/STG can absorb the heat from the hot-stage most efficiently, with rapid and noticeable colour changes, followed by hBN/STG and STG, respectively, indicating that RGO-hBN/STG exhibits the best thermal response under the impact of the constant heat reservoir. Figure 7b displays the temperature–time curves of the corresponding samples. The centre temperature of the sample surface was selected as the observation point. The temperature of RGO-hBN/STG began to stabilise at a time of 140 s under the heating process, about 40 s earlier than the hBN/STG and STG. This means that the temperature of RGO-hBN/STG rises faster than other samples, which is also manifested by the higher slope of the front part of the curve. The stabilised temperature of RGO-hBN/STG, hBN/STG and STG slightly fluctuates at 74.0, 72.1 and 70.6 °C, respectively. All samples stabilise at a constant temperature with elapsed time, which indicates the steady state heat conduction [37]. These results illustrate that the heat absorption performance of RGO-hBN/STG is best among the all samples. To investigate the heat dissipation performance, all samples were removed from the hot-stage and put on the round plate to cool down at ambient condition after heating process. The sensitive colour changes of all samples can be seen in the part of cooling process from Figure 7a. The difference is that the colours of RGO-hBN/STG at the same cooling time is lighter than hBN/STG and STG, indicating better heat release. Their detailed cool-down behaviour can be observed from the cooling curves in Figure 7b. All samples cool down at different heat diffusion rates with time elapsed. They show a relatively large decrease in the surface temperature before 15 s. After this, they exhibit gradual decrease in the temperature variations. Compared with hBN/STG and STG, the RGO-hBN/STG shows much faster decrease with time than hBN/STG and STG. It is worth noting that the surface temperature of RGO-hBN/STG is always lower than hBN/STG and STG at the same cooling time within 300 s. These results show that the heat dissipation performance of RGO-hBN/STG is best in comparison with the hBN/STG and STG. As above, the heat absorption and dissipation performances of all samples take on the same sequence as follows: RGO-hBN/STG > hBN/STG > STG. The two performances demonstrate that RGO-hBN/STG exhibits the best thermal management capability due to its higher thermal conductivity and lower thermal resistance [38,39,40]. Furthermore, the RGO-hBN can improve capacity of heat transmission of silicone thermal grease effectively. Mortazavi et al. [41] carried out multiscale modelling to systematically explore the effective thermal conductivity of graphene and hBN laminates. Their modelling results showed that the heat conduction of graphene and h-BN laminates was affected by the flake size. In agreement with our experimental observations, their multiscale modelling could be developed as an efficient modelling methodology for the assessment of the thermal properties of fabricated structures. Based on multiscale modelling, we will design RGO-hBN with different structural characteristics and tunable thermal conduction properties in a later study.

## 4. Conclusions

One type of novel 3D structure combining with RGO and hBN was fabricated successfully using a rational structural design and investigated as filler for silicone thermal grease. The viscosity of silicone thermal grease filled with 3D RGO-hBN had a much lower value than that of filled hBN nanosheets at the same filler content. The good rheological properties facilitate the processability and constructability of the 3D RGO-hBN-filled silicone thermal grease in practical processing and application. Furthermore, the 3D RGO-hBN enhances the thermal conduction properties of silicone thermal grease, in comparison to hBN nanosheets, which can be ascribed to the introduction of graphene and its phonon-matching structural characteristics. The thermal conductivity enhancement of RGO-hBN/STG conductivity reached 68%, about 1.8 times that of hBN/STG (38%), at a filling ratio of 12 wt.%. Meanwhile, the thermal resistance of the RGO-hBN/STG decreases to a value of 0.138 °C/W from 0.209 °C/W of STG, which is lower than the 0.188 °C/W of the hBN/STG. Importantly, RGO-hBN/STG shows better thermal management capability than STG and hBN/STG during the heating and cooling processes. Hence, the as-fabricated 3D RGO-hBN is a potential candidate as filler for lightweight polymer-based thermal interface materials.
